# Impact of interventions to reduce device-related infections in Indian cancer centre ICUs

**DOI:** 10.1186/cc11778

**Published:** 2012-11-14

**Authors:** S Myatra, JV Divatia

**Affiliations:** 1 Tata Memorial Hospital, Mumbai, India

## Background

Nosocomial infections are important sources of morbidity and mortality in ICUs. Surveillance of nosocomial infection is an integral feature of infection control and quality assurance in hospitals.

## Methods

Our ICU is a nine-bed ICU of a tertiary cancer centre in Mumbai, India. Over a 1-year period, between March 2009 and February 2010 (period 1), we prospectively determined the rates of ventilator-associated pneumonia (VAP) and central line associated bloodstream infection (CLABSI) based on CDC definitions. We also carried out daily process surveillance including compliance to hand hygiene, the ventilator bundle and the central line care bundle. We then introduced the following interventions: routine use of heat-moisture exchanger filters and closed suction systems to reduce VAP, and use of antibiotic-coated central venous catheters to reduce CLABSI. We also reinforced general hygiene practices for all ICU personnel and use of checklists. We then remeasured VAP and CLABSI rates and process compliance over the next 1 year, from March 2010 to February 2011 (period 2).

## Results

In period 1, VAP comprised 61% and CLABSI 35% of all device-related nosocomial infections in the ICU. The CLABSI and VAP rates were 33 and 58 per 1,000 device-days, respectively. VAP and CLABSI accounted for 20% and 7.7% increase in mortality, respectively. The hand hygiene compliance rate was 68% for physicians and 82% for nurses, and compliance with vascular catheter care and ventilator bundles was 65% and 70% respectively. After introduction of the interventions, in period 2, the incidence of VAP was reduced from 58/1,000 ventilator-days to 30.9/1,000 ventilator-days; the CLABSI rate fell to 26.7/1,000 device-days. There was no significant difference in compliance with hand hygiene or the central line and ventilator bundles compared with the previous period.

## Conclusion

Our rates of VAP and CLABSI were much higher than those reported for developing countries and the US NHSN. This may be partly due to the immunosuppressed nature of cancer patients with neutropenia and prior chemotherapy. Our interventions resulted in a significant reduction in the rate of VAP, and to a lesser extent for CLABSI. Process compliance did not change. It is essential to improve process compliance to further reduce the incidence of nosocomial infections in our ICU.

